# Draft Genome Sequence of Methylocella silvestris TVC, a Facultative Methanotroph Isolated from Permafrost

**DOI:** 10.1128/genomeA.00040-18

**Published:** 2018-02-22

**Authors:** Jing Wang, Kan Geng, Muhammad Farhan Ul Haque, Andrew Crombie, Lorna E. Street, Philip A. Wookey, Ke Ma, J. Colin Murrell, Jennifer Pratscher

**Affiliations:** a School of Environmental Sciences, University of East Anglia, Norwich, United Kingdom; b College of Resources and Environmental Sciences, China Agricultural University, Beijing, China; c School of Biological Sciences, University of East Anglia, Norwich, United Kingdom; d School of GeoSciences, University of Edinburgh, Edinburgh, United Kingdom; e Biological and Environmental Sciences, University of Stirling, Stirling, United Kingdom; f Lyell Centre, Heriot-Watt University, Edinburgh, United Kingdom

## Abstract

Permafrost environments play a crucial role in global carbon and methane cycling. We report here the draft genome sequence of Methylocella silvestris TVC, a new facultative methanotroph strain, isolated from the Siksik Creek catchment in the continuous permafrost zone of Inuvik (Northwest Territories, Canada).

## GENOME ANNOUNCEMENT

Methanotrophic bacteria utilize methane as sole carbon and energy sources, thus playing a major role in the global methane cycle (1). They are widespread in the environment, including lakes, rivers, sediments, rice paddies, sewage sludge, forests, and landfill soils (2, 3). All described methanotrophic species belonging to the genus Methylocella (family Beijerinckiaceae) possess a soluble methane monooxygenase (sMMO) that catalyzes the oxidation of methane to methanol, a key methane-oxidizing enzyme that is present in only a subset of methanotrophs (4–6). Methanotrophs of the genus Methylocella do not possess a particulate methane monooxygenase (pMMO), which is present in most other methanotrophs (7). Methylocella species can also utilize multicarbon compounds, including acetate, pyruvate, succinate, ethane, and propane (7, 8). Three species of Methylocella have so far been described, M. palustris, M. silvestris, and M. tundrae (4–6). Type strain M. silvestris BL2 was isolated from an acidic forest cambisol, and its genome sequence was reported previously (9). We now report the isolation and draft genome of a new strain, M. silvestris TVC.

Samples for isolation were taken from a middle hill slope to stream channel transect of the Siksik Creek catchment (68°44′54.5″ N, 133°29′41.7″ W), a tributary of Trail Valley Creek (TVC), Canada (10). After initial enrichment of the soil samples with CH_4_ (18% vol/vol in the headspace), subsamples were transferred to liquid medium and plated repeatedly until the culture was pure.

Genome sequencing of M. silvestris TVC was performed by MicrobesNG (Birmingham, UK) using Illumina HiSeq technology (1,151,332 trimmed reads, 109-fold mean coverage) and assembled, using SPAdes version 3.11.1 (11), into 82 contigs with a genome size of 4,292,072 bp and a G+C content of 62.94%. Annotation was performed using Prokka version 1.12 (12).

Comparative genome analysis revealed that although the 16S rRNA gene sequences of strain TVC and strain BL2 shared 99% nucleotide identity, the similarity of *mmoXYBZDC,* encoding the sMMO, and *mxaF*, encoding methanol dehydrogenase, was 90 to 95% between the two strains. Average nucleotide identity between strains BL2 and TVC was calculated using JSpecies (13), showing 89.80% and 90.59% similarities based on the BLAST algorithm and MUMmer ultrarapid aligning tool, respectively.

Strain TVC used C1 substrates, including methane and methanol, as sole carbon sources. In addition to genes encoding the sMMO and methanol dehydrogenase, genes encoding the tetrahydrofolate (H_4_F)-dependent and tetrahydromethanopterin (H_4_MPT)-dependent pathways of formaldehyde oxidation and those of the serine cycle are present in the genome. Like M. silvestris BL2, strain TVC was a facultative methanotroph and was able to utilize multicarbon compounds, including acetate, ethanol, succinate, and propane. Genes encoding the glyoxylate bypass enzymes isocitrate lyase and malate synthase are present. Also identified were the *prmA*, *prmB*, *prmC*, and *prmD* genes encoding a propane monooxygenase, showing 91%, 84%, 86%, and 86% identities to those of M. silvestris BL2, respectively, enabling growth on propane. Further analyses of the genome and comparison with other strains will lead to a better understanding of the phylogeny and evolution of facultative methanotrophs.

### Accession number(s).

This whole-genome shotgun project has been deposited at DDBJ/ENA/GenBank under the accession number PDZR00000000. The version described in this paper is the first version, PDZR01000000.
